# Supplementary material for: The JNK2-microbiome axis modulates gut barrier integrity through microbial acetate
Source: Gut Microbes. 2026 Apr 8;18(1):2651962. doi: 10.1080/19490976.2026.2651962 (PMC13089537; doi:10.1080/19490976.2026.2651962)
Supplement: Supplementary material — Supplementary captions.docx [file KGMI_A_2651962_SM0020.docx]

**Figure S1: Post-weaning milk-based diets protect from DSS-induced gut inflammation.**

**A**: Composition of macronutrients in different diets: RCD, MBD, MFD, and HFD.

**B**: mRNA levels of proinflammatory cytokines (*Tnfa* and *Ifng)* in the colon tissue were analyzed by RT-qPCR, with Ct values normalized against *Actb*. Dietary groups are indicated.

**C**: Representative images of the colon from DSS- or water-treated mice fed RCD, MBD, MFD, and HFD.

**D&E**: Expression levels of anti-inflammatory cytokines *Il10* and *Tgfβ* were analyzed by RT-qPCR, with Ct values normalized to *Actb* mRNA, in colon tissues of DSS- or water-treated mice fed on RCD, MBD, MFD, and HFD.

**F**: Body weight change in percentage of mice in RCD and MFD fed mice in control and TNBS-treated groups.

**G:** Probability of survival of RCD and MFD-fed mice after TNBS treatment.

**H-I**: Representative images of colon (H), and quantification of colon length (I) in RCD and MFD-fed mice in control and TNBS treated condition.

**J**: Representative H&E-stained colon sections from indicated groups.

**K-L**: Body weight change in percentage of mice in RCD and MFD-fed condition in control and 2.5% DSS treated condition (K), and disease severity score of RCD and MFD-fed mice treated with 2.5% DSS (L).

**M-N**: Representative images of colon (M) and quantification of colon length (N) in control and 2.5% DSS treated condition.

**O**: Representative H&E-stained colon sections from indicated groups.

Data are mean ± SEM, with statistical analysis by two-way ANOVA with Tukey’s multiple comparison test (D, E, F, K, L) and unpaired t test (B and N). P values are shown above the relevant comparisons.

**Figure S2: MBD-fed mice exhibit reduced Immune activation after DSS treatment**

**A**: Schematic illustration of the scRNAseq experimental workflow. 4 weeks after weaning onto MBD or RCD, mice were either subjected to water or 1.5% DSS for 7 days. Colon was dissected, and single-cell suspensions were made. IECs, myeloid cells, T cells, and B cells were isolated, pooled, and subjected to scRNAseq experiment.

**B**: Gating strategy for sorting epithelial, T cell, B cell, and myeloid cells from colon.

**C**: UMAP representation of all detected cells, highlighting epithelial cells, B cells, T cells, and myeloid cells based on markers.

**D**: Dot plot of marker genes highlighting epithelial cells (*Epcam*, *Krt8*, and *Krt18*), B cells (*CD79a*, *Ms4a1*, and *CD19*), T cells (*CD3d*, *CD3e*, and *CD3g*), and myeloid cells (*CD14*, *Itgam*, *Fcgr3*, *Itgax*, *H2-Aa*, and *H2-Ab1*). The color indicates the expression level, and the size of the dots indicates the percentage of cells expressing the gene.

**Figure S3: Milk-based diets maintain gut barrier function even after DSS treatment.**

**A-G**: Dot plots showing expression levels of marker genes for stem cells (A), colonocytes (B), goblet cells (C), cycling TA (D), TA (E), tuft cells (F), and enteroendocrine cells (G) across all epithelial clusters, colored by average gene expression. Dot size indicates the percentage of cells expressing the gene.

**H&J**: The percentage of cells from each experimental group falling into different clusters was plotted as a stacked bar plot. Each bar is standardized to have the same height using the position_fill() argument in ggplot2. Dark blue represents RCD-H2O, green represents RCD-DSS, violet represents MBD-H2O, and yellow represents MBD-DSS.

**I**: Dot plots showing expression levels of inflammatory genes across epithelial cell clusters. Color intensity indicates the average expression level, and the size of the dot indicates the percentage of cells expressing the gene.

**Figure S4: MBD-fed mice exhibit reduced Immune activation after DSS treatment.**

**A**: UMAP representations and donut plots showing the distribution of B cells from different experimental groups across different cell types. The experimental groups are RCD-H2O (RCD on water), RCD-DSS (RCD on 1.5% DSS), MBD-H2O (MBD on water), and MBD-DSS (MBD on 1.5% DSS). The percentage of cells belonging to some of the cell types is shown next to each donut plot.

**B**: UMAP representation and donut plot showing the distribution of T cells across different clusters. The percentage of cells belonging to some of the cell types is shown next to the donut plot.

**C**: UMAP plots colored by the expression of T cell marker genes (*Cd4, Cd8a, Cd8b1, Ifng, Gzma,* and *Cd69*). The intensity of the color indicates the expression levels, as shown in the color scale given with each plot.

**D**: Dot plot showing expression of various T cell markers, highlighting different subsets such as Th1, Treg, Th2, and Th17 cells. The color indicates the expression level, and the size of the dots indicates the percentage of cells expressing the gene.

**E**: The percentage of cells from each experimental group falling into different clusters was plotted as a stacked bar plot. Each bar is standardized to have the same height by using the position_fill() argument in ggplot2. Dark blue represents RCD-H2O, green represents RCD-DSS, violet represents MBD-H2O, and yellow represents MBD-DSS.

**F**: UMAP representation of all myeloid cells (*Cd14*, *Itgam*, *Fcgr3*, *Itgax*) showing 22 clusters.

**G**: Donut plots show the distribution of myeloid cells from different experimental groups across different clusters. The percentage of cells belonging to some of the clusters is shown next to each donut plot.

**H-J**: Dot plot showing expression of various monocyte (H), macrophage (I), and cDC2 (J) marker genes. The color indicates the expression level, and the size of the dots indicates the percentage of cells expressing the gene.

**K**: UMAP plots colored by the expression of cytotoxic NK cell marker genes (*Klrk1/NKG2D*, *Cd7*, *Cd27*, *Gzma*, *Gzmb*, *Prf1*, *Fasl*, and *Tnfsf10*). The intensity of the color indicates the expression levels, as shown in the color scale given with each plot.

**L**: Bar plot showing -log10(adjusted p-value) of GO terms enriched in cluster 0 of myeloid cells. The color indicates the number of genes differentially expressed in cluster 0 that overlap with the indicated GO term.

M: Dot plot showing average expression and percentage of cells expressing indicated genes across clusters.

**Figure S5: Milk-based diets elicit protective effects against gut inflammation by activating the JNK2 pathway in the epithelial cells.**

**A**: Heatmap showing the regulome expression calculated using the SCENIC package.

**B**: mRNA levels of AP-1 transcription factors (*c-Jun, Junb, and Atf3)* in the IECs were analyzed by RT-qPCR, with Ct values normalized against *Actb*. Dietary groups are indicated.

**C**: Network graph showing the interaction between transcription factors identified in the SCENIC analysis and gut barrier and AMP genes analyzed by StringDB.

**D**: p-p38 target genes associated with inflammation (*Cox2* and *Ptges2*) analyzed by RT-qPCR, with Ct values normalized to *Actb*, in colon tissues of DSS- or water-treated mice, fed RCD, MBD, MFD, and HFD.

**E**: Western blot analysis for p-JNK and JNK levels in IECs isolated from JNK inhibitor II treated mice fed on RCD. Samples were collected at 6 and 24 hrs post-inhibitor treatment.

**F**: Representative images of colon, DSS- or water-treated, with or without JNK inhibitor II. Diet groups are indicated.

**G**: Representative images of H&E-stained colon tissue sections of RCD and MFD-fed mice treated with DSS or water and subjected to JNK inhibition or vehicle treatment.

**H**: Gut-barrier-associated genes *Cld7* and *Hnf4a* analyzed by RT-qPCR, with Ct values normalized to *Actb*, in colon tissues of DSS- or water-treated mice with or without JNK inhibition, fed on MFD.

**I**: p-JNK and JNK levels were analyzed in IECs from mice fed on RCD and MFD, isolated 7 days after p-JNK inhibition. Representative blot (left panel) and densitometric quantification (right panel) are shown.

**J**: Western blot analysis for Cyclin D1 levels in IECs isolated from RCD, MBD, MFD, and HFD-fed mice.

**K**: Representative images of Ki67 immunohistochemical staining in colon tissue from RCD, MBD, MFD, and HFD-fed mice (left panel) and their quantification (right panel).

**L**: Apoptosis-related genes (*Puma* and *Noxa*) analyzed by RT-qPCR, with Ct values normalized to *Actb*, in colon tissues of DSS- or water-treated mice, fed RCD, MBD, MFD, and HFD.

**M**: Western blot analysis for cleaved caspase-3 was done in IECs isolated from RCD, MBD, MFD, and HFD-fed mice. Representative blot (left panel) and densitometric quantification (right panel) are shown.

**N**: Western blot analysis for MKK7 was done in IECs isolated from RCD, MBD, MFD, and HFD-fed mice. Representative blot (left panel) and densitometric quantification (right panel) are shown.

**O**: Western blot analysis for MKK4 was done in IECs isolated from RCD, MBD, MFD, and HFD-fed mice. Representative blot (left panel) and densitometric quantification (right panel) are shown.

Data are mean ± SEM, with statistical analysis by two-way ANOVA with Tukey’s multiple comparison test (D, H, and I) and one-way ANOVA with Tukey’s multiple comparison test (B, K, L, M, N, and O). p values are given above relevant comparisons.

**Figure S6: Microbiota depletion and transfer reveal diet-dependent regulation of epithelial barrier and inflammatory responses**

**A**: LB agar plate showing fecal microbiota depletion in antibiotic-treated mice. 20 mg of fecal matter was resuspended in 1 ml PBS and diluted 1/4 fold and 1/8 fold and spotted on the plate. Each black circle represents a sample from a mouse. Representative qPCR amplification curves of bacterial 16S rRNA gene in control and antibiotic-treated (ABX) groups are given in the right panel.

**B-D**: RT-qPCR analysis of expression of *Hnf4a* (B), transcripts of antimicrobial peptides Reg3b and Lypd8 (C), and gut barrier genes (*Tjp1*, *Cld7*, *Ocln*, and *Muc2*) (D) in IECs of ABX-treated mice fed on RCD, MFD, and HFD.

**E**: Western blot analyses of IECs isolated from mice with or without antibiotic (ABX) treatment, fed RCD, MFD, and HFD, showing p-p38 and total p38. The panel on the right-hand side shows densitometric quantification.

**F**: RT-qPCR analysis of expression of inflammatory genes (*Cox2*, *Ptges2*, and *Tnfa*).

**G**: Representative qPCR amplification curves of bacterial 16S rRNA gene before PEG treatment and 6 h after PEG.

**H.** Representative images of colons from no DSS controls, DSS-treated mice without FMT, and DSS-treated mice with FMT (MFD-RCD, RCD-MFD, MFD-MFD).

**I.** Representative H&E-stained colon sections from indicated groups. Scale bar, 150 µm.

Data are mean ± SEM, with statistical analysis by two-way ANOVA with Tukey’s multiple comparison test (B, C, D, E and F). p values are given above the relevant comparisons.

**Figure S7: Dubosiella Newyorkensis, enriched in the milk-based diet groups, provides gut protection.**

**A**: Microbial-diversity indices [Shannon and inverse Simpson] of fecal microbiome isolated from mice fed on RCD, MBD, MFD, and HFD.

**B**: Venn diagram illustrating the common and unique species in the microbiomes of RCD, MBD, MFD, and HFD-fed mice.

**C-E**: Volcano plots showing differentially enriched species across the conditions: MFD vs RCD, MBD vs RCD, and HFD vs RCD. The size of the dots indicates the abundance of the species

**F**: Schematic illustration of the experimental workflow for *D. newyorkensis* treatment.

**G-I**: RT-qPCR analysis of proinflammatory cytokines, *Tnfa, Il1b* (G) antimicrobial peptides Reg3b and Lypd8 (H), and gut barrier genes (*Cldn7, Tjp1, Ocln, Muc2,* and *Hnf4a*) (I) in colon tissues of D. newyorkensis treated mice.

Data are mean ± SEM, with statistical analysis by two-way ANOVA with Tukey’s multiple

comparison test (G, H, and I) and DESeq2 Padj<0.01 and absolute Log2 foldchange of>1 (C, D and E). *p values are shown above the relevant comparisons.

**Figure S8: Acetate induces the JNK2 pathway in the colonic epithelium to enhance gut barrier function.**

**A**: Quantification of fecal acetate, propionate, and butyrate using GC-MS in RCD, MFD, and HFD fed mice groups.

**B**: Levels of propionate and butyrate in the fecal matter of control and *D. newyorkensis*-treated mice.

**C-D**: Representative images of colons from control or acetate-treated mice with and without 1.5% DSS challenge (C) and quantification of the colon lengths post DSS insult (D).

**E-F**: RT-qPCR analysis of proinflammatory cytokines (*Tnfa, Il1b,* and *Ifng*) (E) and gut barrier genes (*Ocln*, *Muc2*, and *Hnf4a*) (F) in colon tissues of control or acetate-treated mice with or without 1.5% DSS challenge.

**G**: Western blot analyses of IECs isolated from control and acetate-treated mice showing p-p38 and total-p38 levels. Densitometric quantitation is given in the right panel.

**H**: RT-qPCR analysis of antimicrobial protein mRNAs (*Reg3b* and *Lypd8*) in colon tissues of control or acetate-treated mice with or without 1.5% DSS challenge.

**I**: Western blot analyses of IECs isolated from vehicle and GPR43 inhibitor, GLPG0974-treated mice, showing p-JNK and total-JNK levels. Densitometric quantitation is given in the right panel.

**J**: Representative immunoblot showing p-JNK and total JNK levels in Caco-2 cells treated with increasing concentrations (0–50 mM) of KCl or potassium acetate (K-acetate).

Data are presented as mean ± SEM. Statistical analysis was performed using one-way ANOVA with Tukey’s multiple comparison test (A), two-way ANOVA with Tukey’s multiple comparison test (D–F, H, I), and unpaired two-tailed Student’s t-test (B, G). p values are given above each relevant comparison.

**Figure S9: Timing of MFD exposure governs protection, microbiome composition, and metabolite profiles.**

**A-C**: Disease severity, body weight (%), and colon length following first (DSS I) and second (DSS II) DSS cycles in RCD(I&II), MFD(I&II), and RCD(I)-MFD(II) groups. Colon length was measured after euthanising the mice at the end of DSS II.

**D**: Schematic of dietary switching strategy prior to DSS treatment.

**E-G**: Disease severity, body weight (%), and colon length in RCD, MFD, and MFD-RCD groups.

**H-J**: Disease severity, colon length, and representative colon images in RCD, RCD(1wk)-MFD(3wk), and MFD groups.

**K-M**: Alpha diversity indices (Shannon and Inverse Simpson indices), species-level relative abundance, and fecal short-chain fatty acid levels in indicated groups.

Data are presented as mean ± SEM; Statistical analysis was performed using two-way ANOVA with Tukey’s multiple comparison test (A-C, E-I), and unpaired two-tailed Student’s t-test (K, M). p values are indicated on the relevant comparisons.

**Table S1:** Macronutrient composition of the diets used in this study.

**Table S2:** Proportion of different cell types in the epithelial compartment as determined by scRNAseq, shown in Figure 2B.

**Table S3:** List of genes used to calculate module scores.

**Table S4**: List of chemicals and reagents used in the study.

**Table S5**: List of antibodies used in the study.

**Table S6**: List of commercially available kits used in the study.

**Table S7**: Use of live organisms used in the study.

**Table S8**: List of rodent diets used in the study. Details of the ingredients of each diet is given in supplementary dataset 2.

Supplementary Dataset 1: Abundance of different species identified using 16s rRNA sequencing.

Supplementary Dataset 2: GC/MS data from mice treated with *D. newyorkensis* or with different diets.

Supplementary Dataset 3: Detailed composition of diets used in the study
