# Supplementary figures and images for: The JNK2-microbiome axis modulates gut barrier integrity through microbial acetate
Source: Gut Microbes. 2026 Apr 8;18(1):2651962. doi: 10.1080/19490976.2026.2651962 (PMC13089537; doi:10.1080/19490976.2026.2651962)

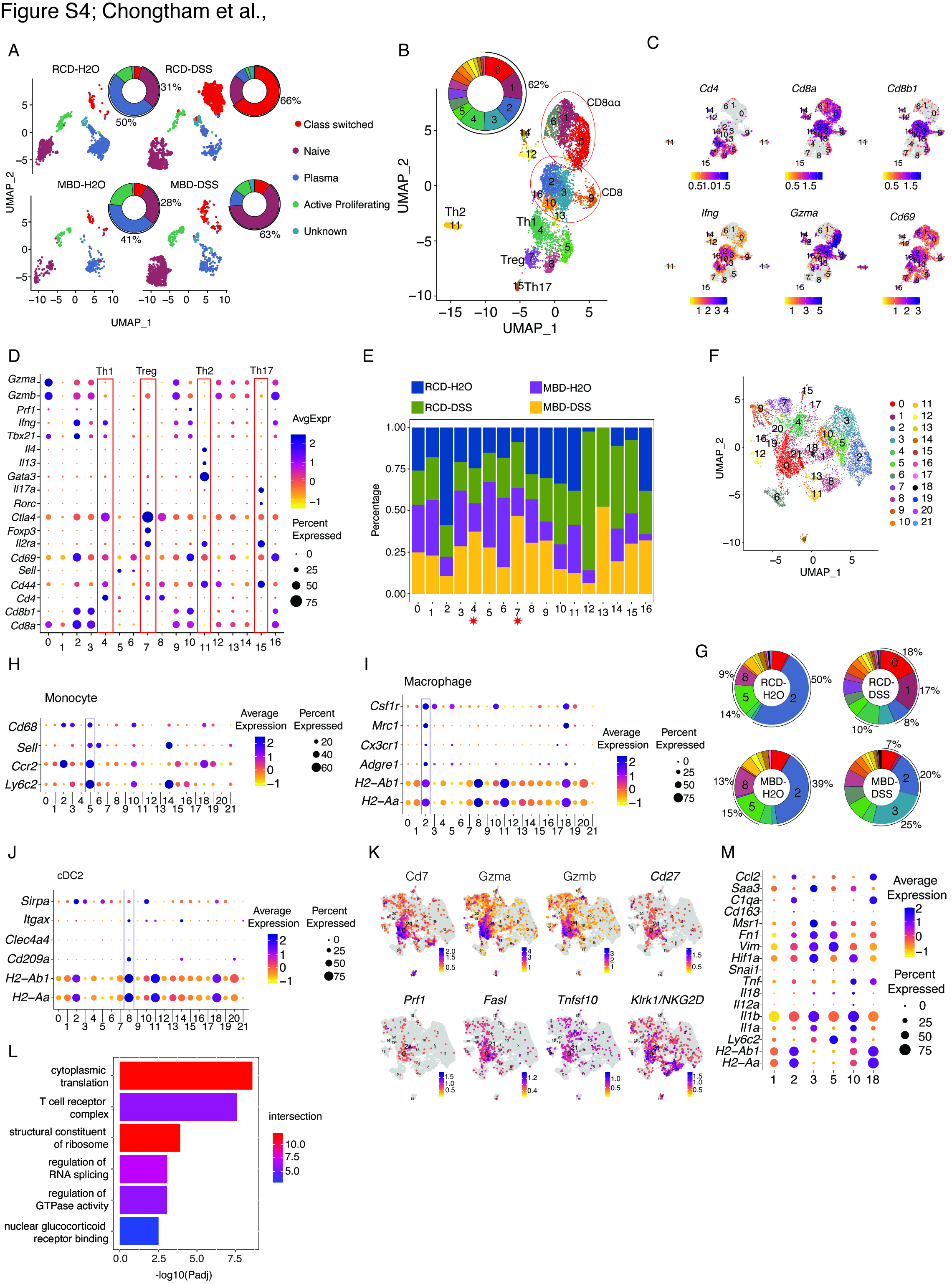

Supplement: Supplementary material — Supplementary Figure.zip [file KGMI_A_2651962_SM8472.zip › Figure/Figure S4.tif]

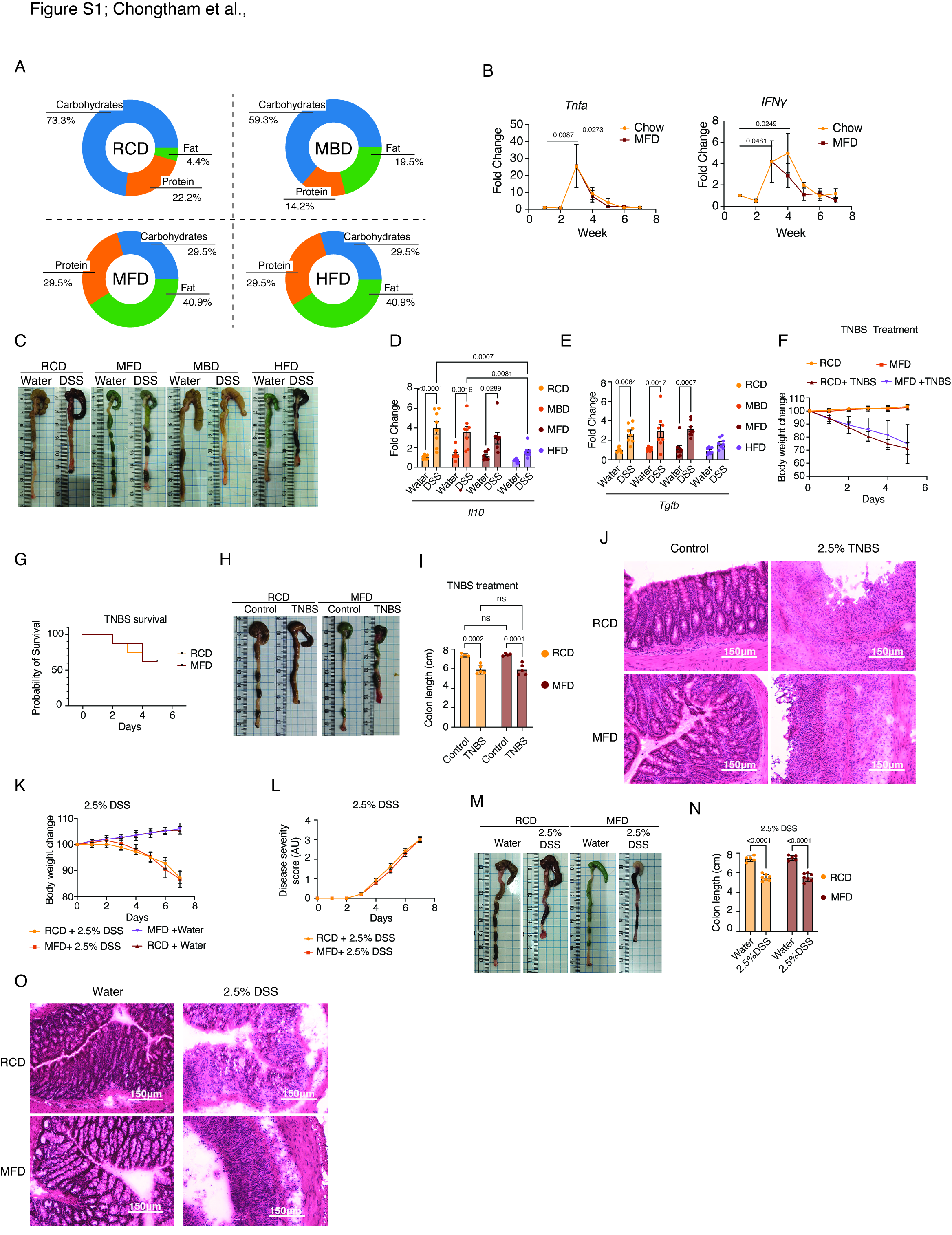

Supplement: Supplementary material — Supplementary Figure.zip [file KGMI_A_2651962_SM8472.zip › Figure/new_fig_S1_v2.tif]

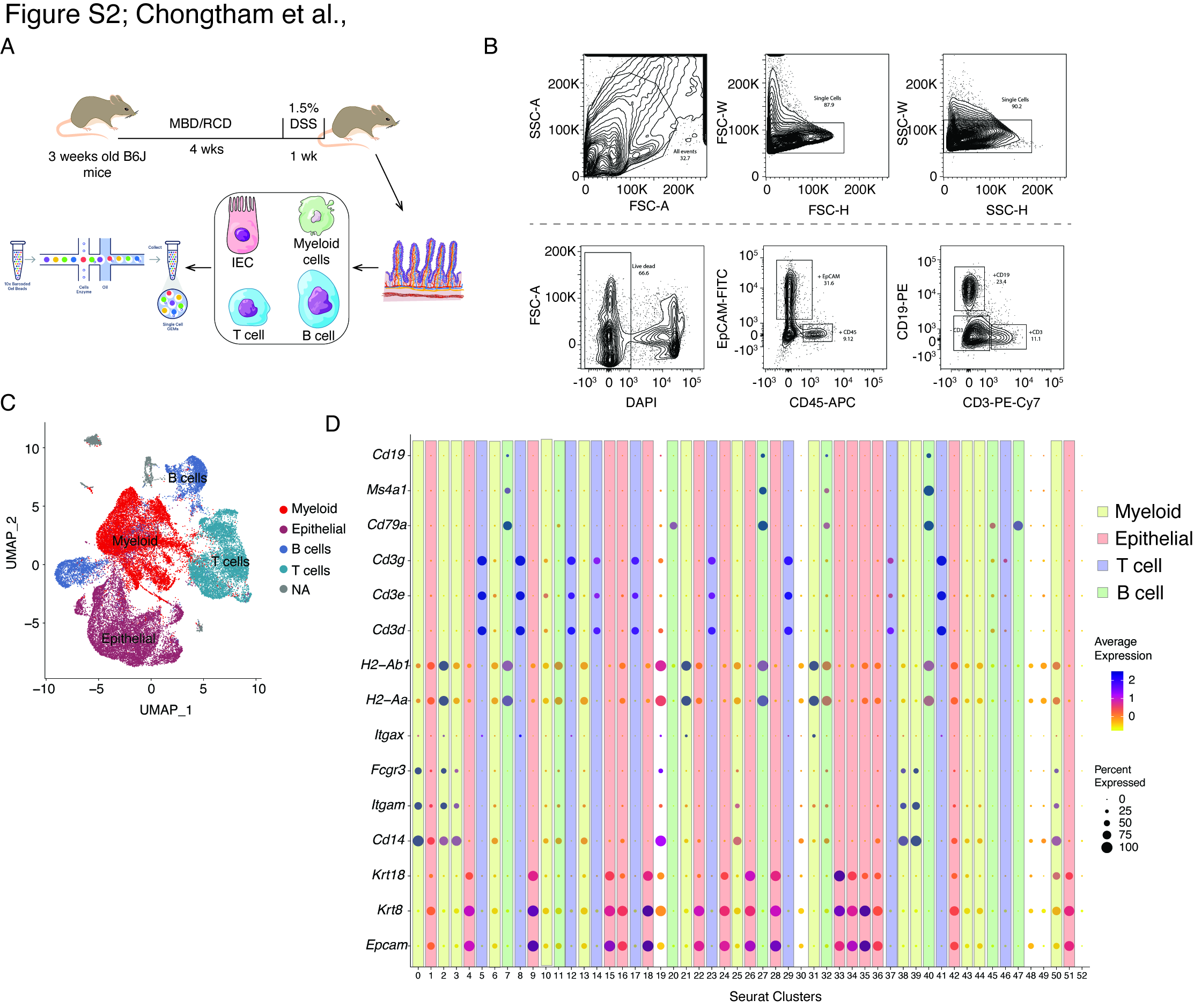

Supplement: Supplementary material — Supplementary Figure.zip [file KGMI_A_2651962_SM8472.zip › Figure/New_Fig_S2.tif]

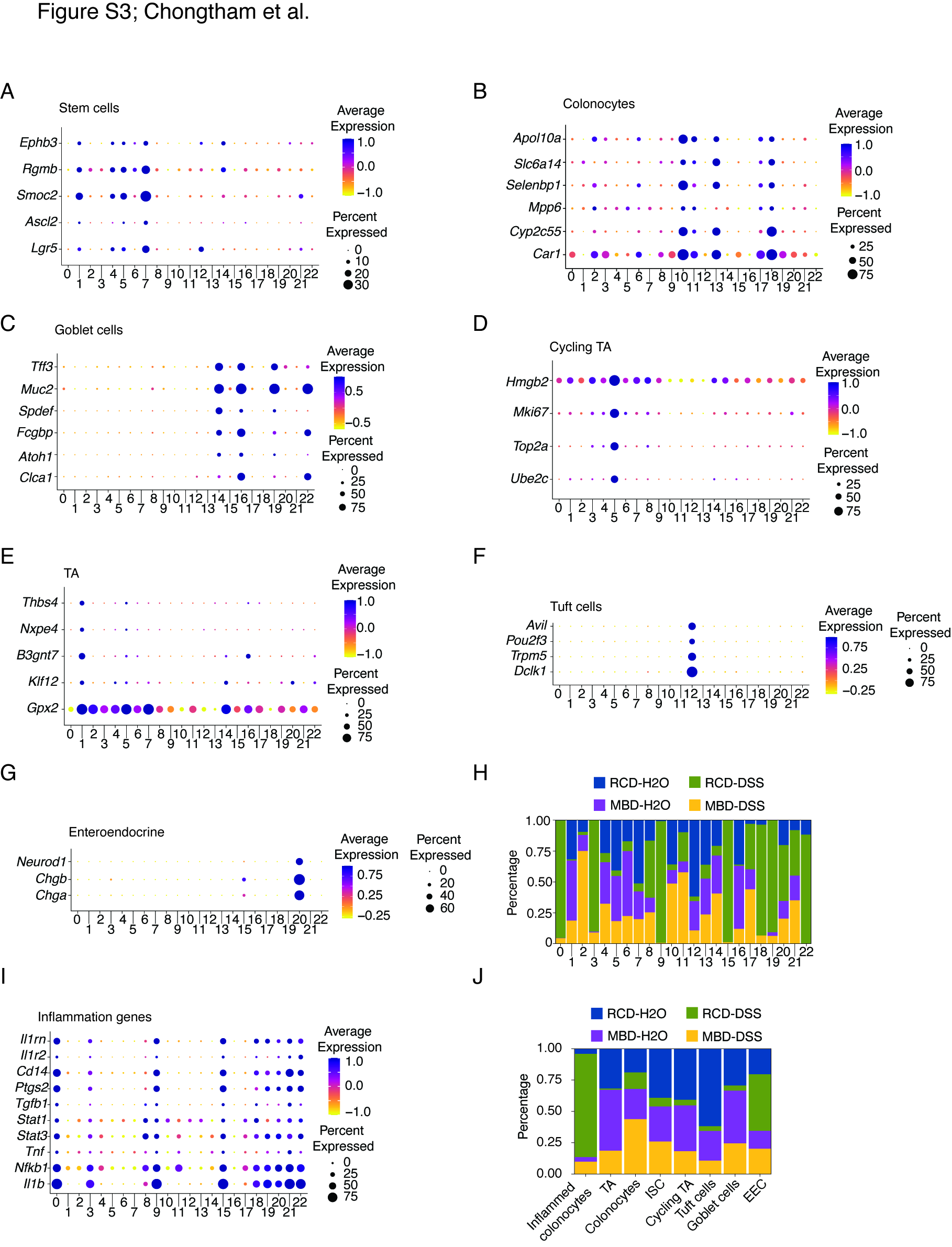

Supplement: Supplementary material — Supplementary Figure.zip [file KGMI_A_2651962_SM8472.zip › Figure/new_fig_S3.tif]

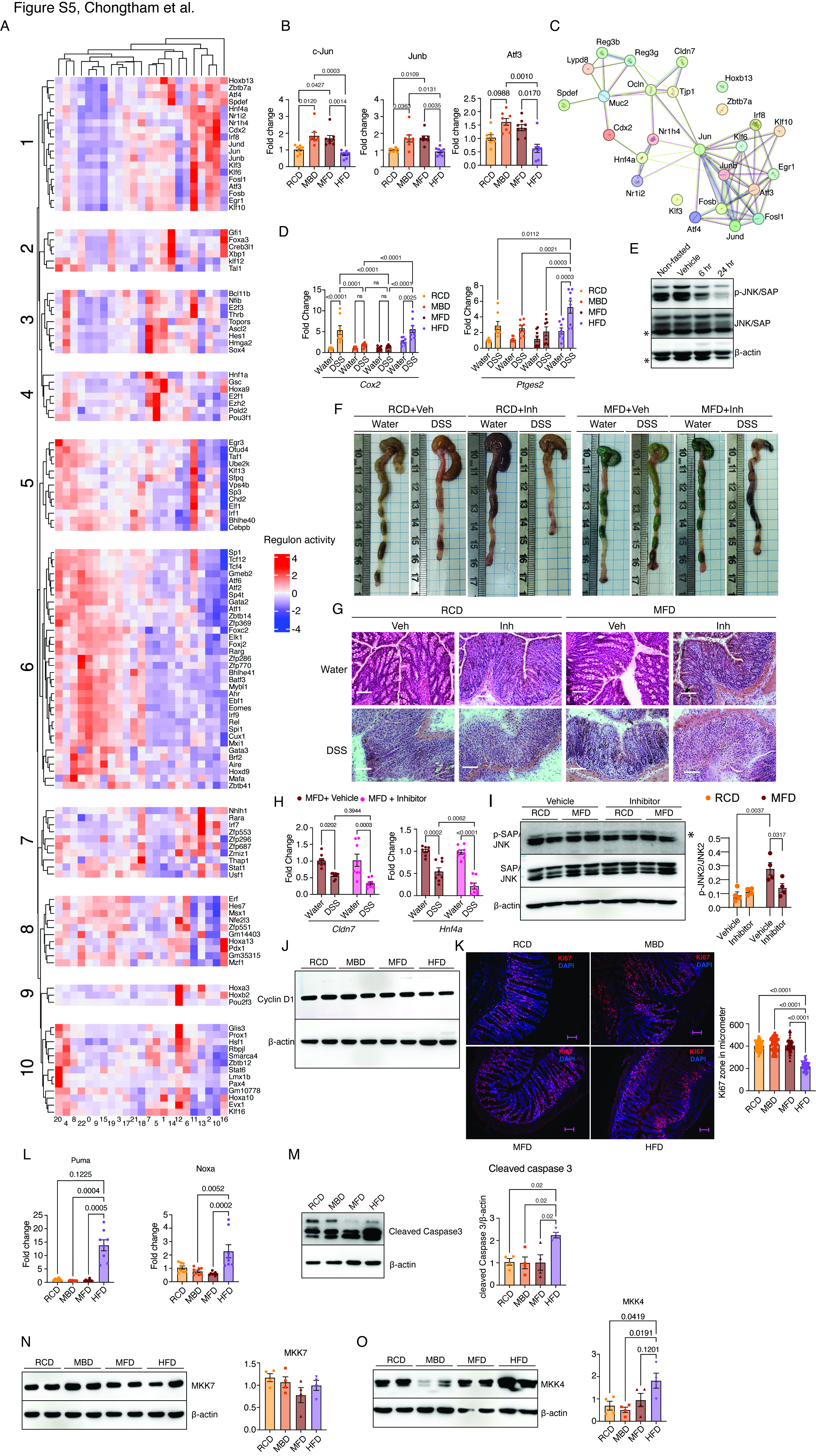

Supplement: Supplementary material — Supplementary Figure.zip [file KGMI_A_2651962_SM8472.zip › Figure/New_Fig_S5.tif]

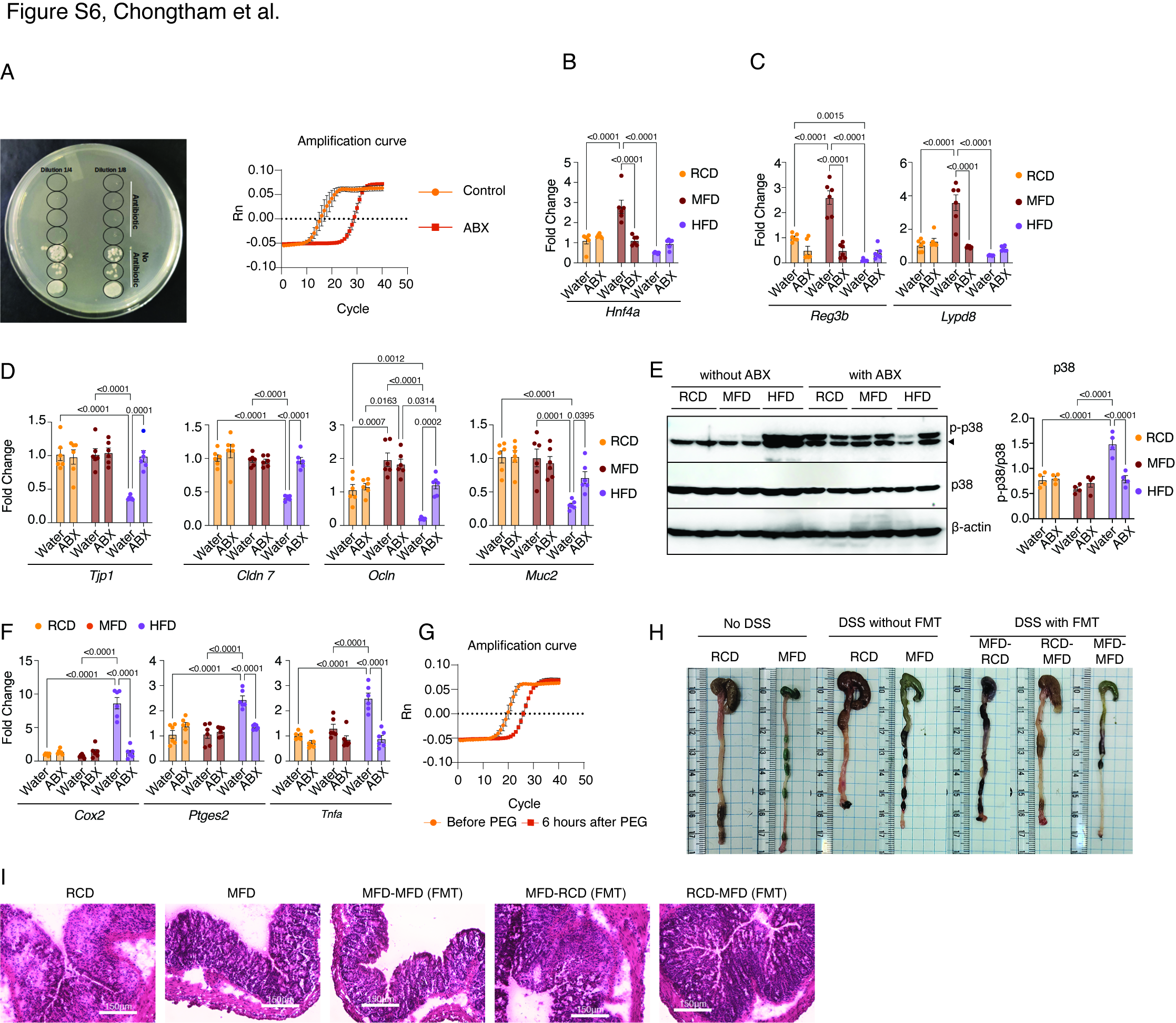

Supplement: Supplementary material — Supplementary Figure.zip [file KGMI_A_2651962_SM8472.zip › Figure/New_Fig_S6c.tif]

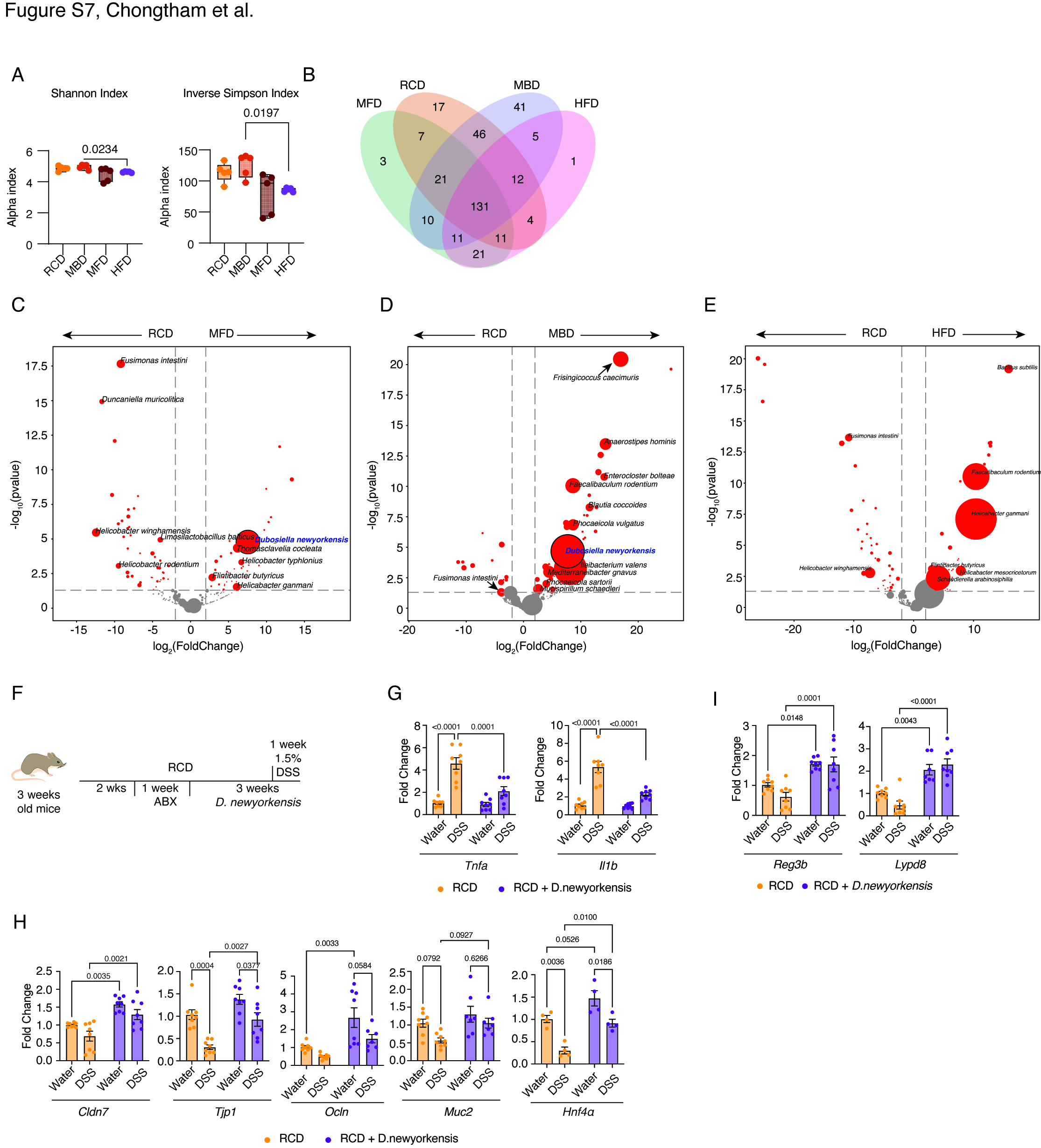

Supplement: Supplementary material — Supplementary Figure.zip [file KGMI_A_2651962_SM8472.zip › Figure/New_Fig_S7.tif]

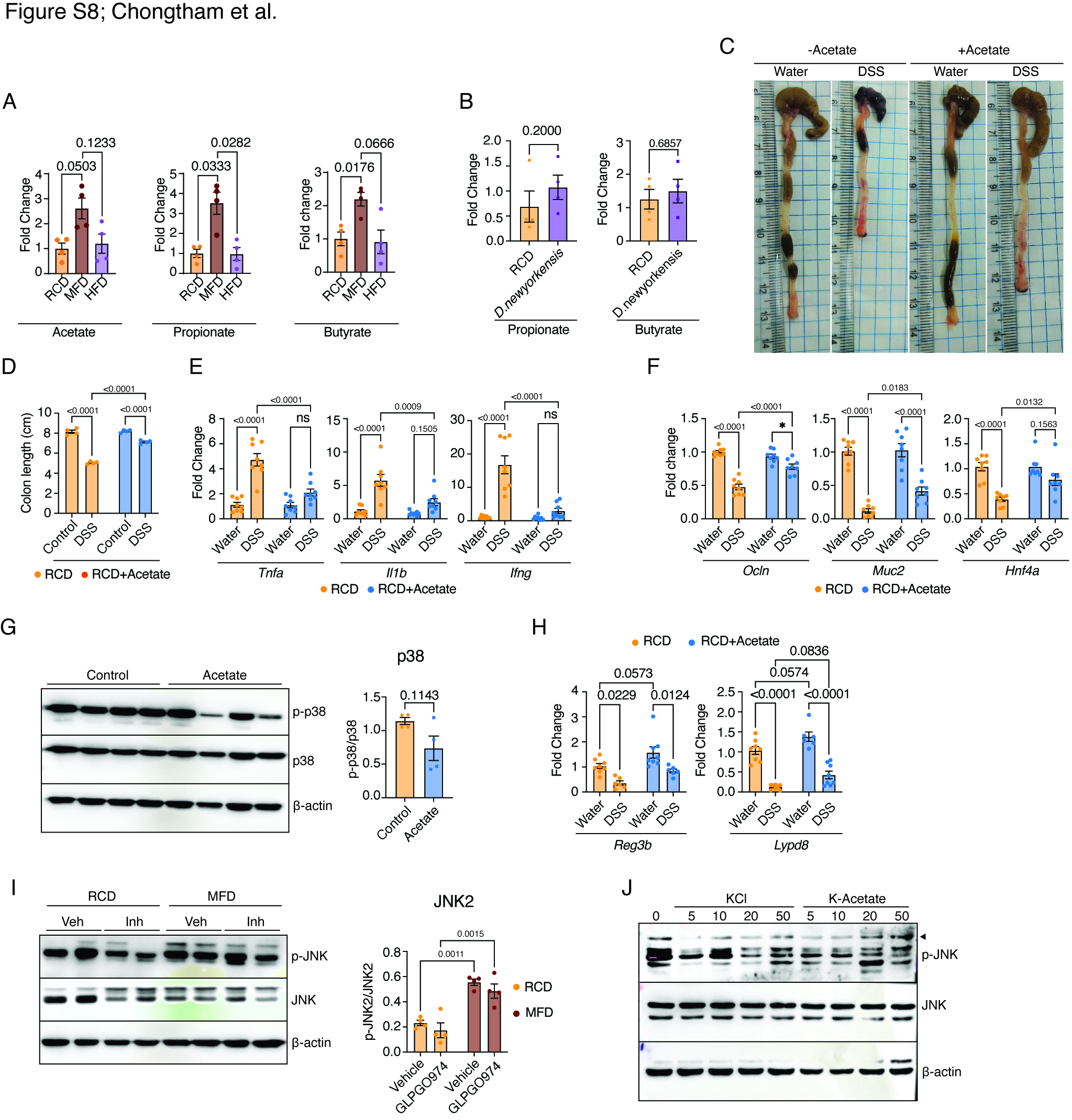

Supplement: Supplementary material — Supplementary Figure.zip [file KGMI_A_2651962_SM8472.zip › Figure/New_Fig_S8.tif]

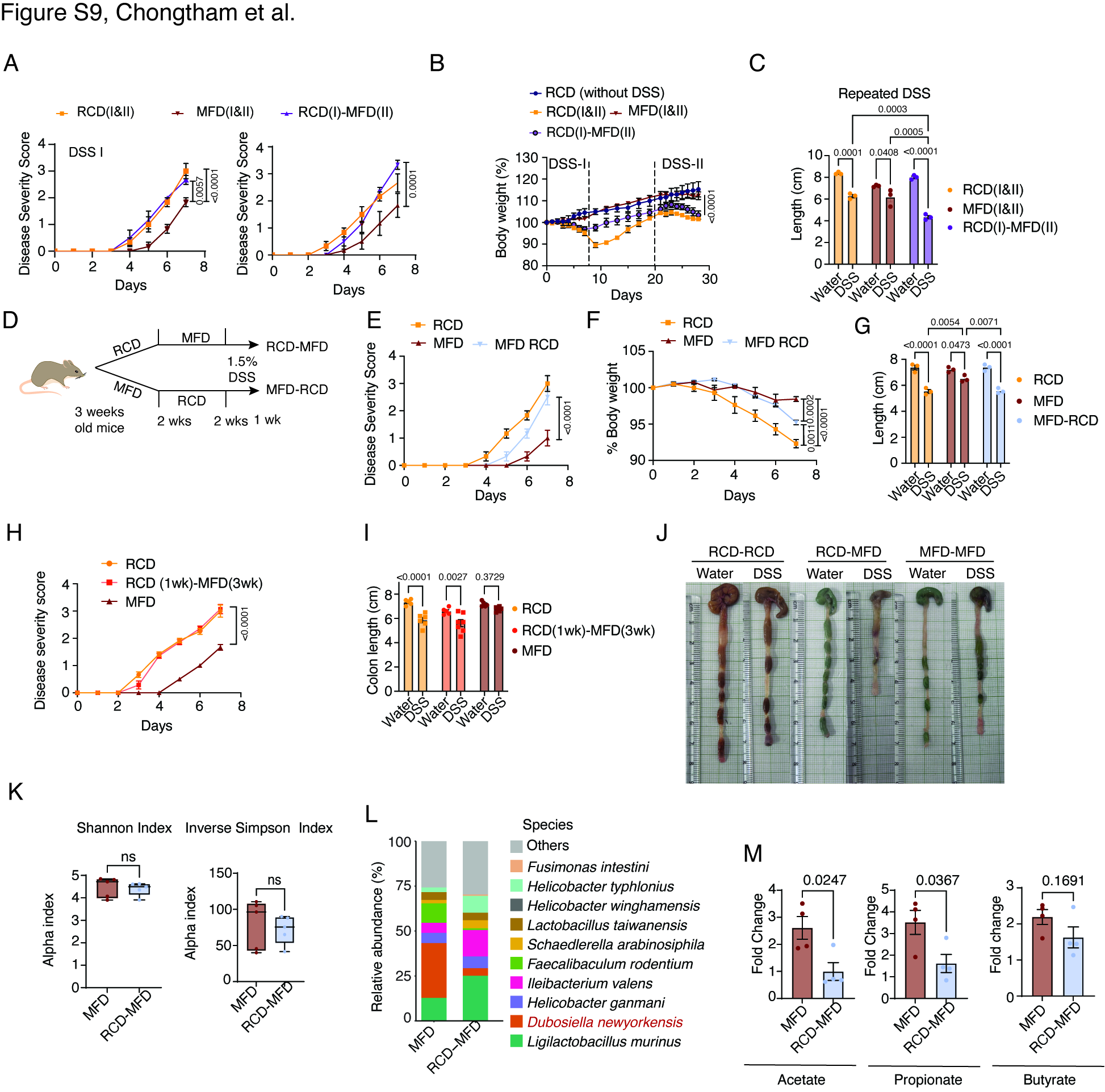

Supplement: Supplementary material — Supplementary Figure.zip [file KGMI_A_2651962_SM8472.zip › Figure/New_Fig_S9.tif]
